# Perception of health care students towards lectures as a teaching and learning method in the COVID era - A multicentric cross-sectional study from India

**DOI:** 10.12688/f1000research.110100.1

**Published:** 2022-06-16

**Authors:** Vijay Pratap Singh, Anand Ramakrishna, Neloy Sinha, Bidita Khandelwal, Nitin Joseph, Purnima Barua

**Affiliations:** 1 Department of Physiotherapy, Kasturba Medical College, Mangalore, Manipal Academy of Higher Education, Manipal, India; 2 Department of Respiratory Medicine & Medical Education, Kasturba Medical College, Mangalore, Manipal Academy of Higher Education, Manipal, India; 3 Department of Dermatology, College of Medicine and JNM Hospital, West Bengal, Kalyani, India; 4 Department of Medicine, Sikkim Manipal Institute of Medical Sciences, Sikkim Manipal University, Gangtok, India; 5 Department of Community Medicine, Kasturba Medical College, Mangalore, Manipal Academy of Higher Education, Manipal, India; 6 Department of Microbiology, Jorhat Medical College, Jorhat, Assam, India

**Keywords:** classroom lectures, COVID era, perception, students, health profession

## Abstract

The sudden precipitation of the pandemic forced undergraduates to take refuge at home, deserting the campus. Consequently, the age-old classroom in person teaching-learning (T-L) method shifted and lessons had to be conducted online. In previous decades, archetypical classroom lectures survived a lot of criticism in the face of the quasi-passive nature of T-L  methodology. There are very few studies that reflect undergraduate students' perceptions of lectures. This study aimed to evaluate undergraduate students’ perceptions of lectures using an online questionnaire with 13 items, which was circulated to undergraduate students of medical, physiotherapy, and nursing courses in three settings at different locations of private and public health schools. There was a total of 877 responses. The surveyed students were in favor of lectures and considered them indispensable for undergraduate learning. They preferred it as a kind of organized learning through the teacher’s own experiences. Our study suggests that it is not the ‘lecture’ that requires mending but possibly teachers require better training, application of effective audio-visual aids, and innovative techniques to sustain students' interest in the class.

## Introduction

The lecture was an integral part of the revered teaching-learning method since the time of *Gurukul* *.* ^1^ It might have morphed internally to tackle the ever-increasing demands of modern educational policies and pressures but it cannot be completely replaced in the name of innovative teaching methods. ^2^

Without being prejudiced against the tech-savvy tribe, we must revisit the basics. Is India a technology-supported country at the grassroots level? Do we have adequate broadband strength in remote villages, hilly areas, and in low economic setups?

In India broadband strength is a concern for many residents in hilly areas and in low economic setups. Despite knowing the answers to the above questions, in this “COVID-19 lockdown” phase, the media highlighted the poor access of rural students and their inability toward 100% participation in the online teaching-learning opportunities. ^3^ A sudden move toward digital technology will undermine the underprivileged section while working in favor of the urban learning fraternity. ^4^^,^^5^

None of the methods are good or bad but should be evaluated from the perspective of the situational fulcrum. A search of literature revealed a scarcity of methodical in-depth studies that showed students’ perceptions of lectures from a global perspective. While earlier authors emphasized the rebuilding of a new curriculum in health/medical education, which could be created through digital and internet media, they also undermined the effectiveness of classroom lectures. ^6^ We take this opportunity to find out students’ perception toward lectures, a method that is time tested but had to undergo and withstand strong criticisms from the penmanship and propaganda of educationists of the past who wanted to devote their time in publication at the cost of classroom teaching. ^6^^,^^7^ They considered classroom teaching as sacrificial and were in favor of emerging technologies. ^7^^,^^8^ Most studies that criticized lectures were either viewpoints of teachers or policymakers to stress their side of the story. ^9^^,^^10^ There is scarcity of data about the viewpoints of the students and what they think about lectures. The COVID-19 era is the right time to explore students’ perceptions of lectures, due to the rapid shift away from this technique, and we aim to do this through a structured set of questionnaires. This study operationally defines lectures as traditional classroom ones, which are large group teaching methods, face-to-face, and in accordance with the publications by Brown ^2^ and Harden. ^11^ Brown and Manogue ^2^ focused on the importance of a lecture that cannot be replaced by any other teaching-learning method, its limitations, different methods, how learning happens through lectures, and skills required for a good interactive one. Harden and Crosby ^11^ explained how a lecturer should emulate the role of a teacher, and the authors mentioned 12 roles of a teacher that cannot be seen in a different canvas; rather, it overlaps with the lecturer model. A lecturer is a teacher and his interaction with students is not just an hourly lecture but a holistic role to be part of their learning and making their learning environment safe. ^2^^,^^10^ The sudden shift of policy makers’ attention to research pushed lectures to a somewhat lower pedestal and the time dedicated to lecturing and mentoring got diverted to research. Students’ views were hardly included, and everything was left in the name of self-directed learning. ^12^^,^^13^ It is a major method of teaching and learning for post-graduates, but its applicability in undergraduate and preclinical days should be evaluated with caution. If students are not sensitized primarily to an entirely new topic, self-directed learning can prove ineffective. ^14^ There is no roadmap provided to them. In such cases, lectures are of great use, these can give a blueprint for learning a topic through the experiences of the lecturer and students can build upon it by self-directed learning as per their ability and interest in line with the competencies required. ^15^

To address some of the concerns mentioned in this section, this study aims to evaluate students’ perceptions of lectures in different settings, courses, and among private and state-supported health care institutions.

## Methods

The study was approved by the institutional ethics committee of Kasturba Medical College, Mangalore registration number ECR/541/Inst/KA/2014/RR-20 and reference code IECKMCMLR-08/2020/223. The study was carried out in accordance with relevant guidelines and regulations in ethics approval. All participants were asked for written informed consent for both collection of their data and for publication of this data, those who did not give this consent were not included in this study. Participants were students from the first to final year enrolled in medical, nursing, or physiotherapy courses from the Sikkim Manipal Institute of Medical Sciences (SMIMS), Gangtok; Kasturba Medical College (KMC), Mangalore; and Jorhat Medical College (JMC), Assam. It was a cross-sectional, exploratory, online survey that was implemented online through a questionnaire. We aimed for complete enumeration, blinded, and voluntary, without asking the identity of the students. This was to avoid bias.

This questionnaire based study was done during the first wave of COVID-19. We conducted the study in two phases. In phase I, we developed and validated the questionnaire, and in phase II, we measured the students’ perceptions of lectures during the COVID-19 period when most teaching happened through the online mode.

To the best of our knowledge we could not retrieve a questionnaire which measured students perception after sudden shift from classroom teaching-learning to internet based online teaching. Therefore, there was a challenge to develop a questionnaire which can measure the intended objective of this study. We used the keywords “lectures”, “class room lectures”, “large group teaching”, “Students perception”, “student experiences” and combined it with the BOOLEAN operator “AND.” We searched the following databases: PubMed **(MEDLINE, RRID:SCR_002185)**, Cochrane **(Cochrane Library, RRID:SCR_013000)**, Web of Science **(Clarivate Analytics, RRID:SCR_017657)** for articles published till August 2020 and retrieved 52 studies. After screening the articles, we included some studies and experts’ opinion to develop items for the questionnaire.

The initial questionnaire had 20 items. Initial items for the study were generated by our team of ten teaching faculties from medical education unit with three rounds of brainstorming meetings and consensus-building. Initial questionnaire was preliminary and was based upon faculties experiences, literature search and informal discussions. Then an initial item pool was created and the items were tested with 30 students from medical, physiotherapy, and nursing courses to reach a consensus. These students were class representatives in academic or co-curricular committees. These students were selected because they have maximum interaction with students and are seen as representatives for what students thought about sudden shift of on-campus lecture hall classes to online mode. The questionnaire was sent to these students as a Google Form (Google, Google Forms) but meetings were held through online video modes to derive final set of questions. ^34^ In pilot testing students were asked to rate each question on a Likert scale ranging from 1 to 5 (where 1=not at all relevant and 5=extremely relevant). Open feedback was also taken on any items not included, wording and clarity of the questions. For item validation responses were also categorized into dichotomy as agree, if respondent scored 3 or more. Item-content validity index (I-CVI) and scale-content validity index (S-CVI) were calculated for the item pool. The I-CVI was calculated by dividing the number of agreed participants by the total number of participants’ responses according to the dichotomous response scale. ^16^ The S-CVI was calculated by taking the average I-CVI and dividing it by the total number of items. The acceptable agreement for I-CVI was 0.78 or higher, and the acceptable agreement for S-CVI was 0.80 or higher. The research team used I-CVI and any open responses in the survey to guide them in modifying, removing, or replacing the items.

A final set of questions with 13 items was chosen, as shown in Table 1. The reliability of the data collection tool was calculated using Cronbach’s alpha value and was found to be 0.72 indicating good internal consistency. ^17^

**Table 1.** The 13 items of the questionnaire and Likert scale ranging from SA (Strongly agree-5), A (Agree-4), N (Neutral-3), D (Diasagree-2) to SD (Strongly disagree-1).

Question number.	Items	(5) Strongly agree	(4) Agree	(3) Neutral	(2) Disagree	(1) Completely disagree
1	I miss lectures when there is long gap in classroom teaching, for example during lockdown due to COVID-19.					
2	I genuinely miss lectures.					
3	I am happy at least online teaching is available.					
4	Online teaching cannot replace classroom teaching and lectures though.					
5	It is the quality of the teacher who carries the lecture in a good way, I am always interested in attending good lectures.					
6	I bunk some of the lectures because I don’t find them interesting. They are either reproducing the book or a confused teacher with unorganized teaching matter.					
7	I find lectures are not useful and they can be replaced with technology like video-assisted or self-study.					
8	I think I eagerly wait for some classes because of Lecturer’s ability to teach.					
9	I think online distance classes are enough and lecture hours can be replaced with them.					
10	I genuinely agree lectures are the most important method of teaching-learning.					
11	It depends on the ability of the teacher to impart lectures effectively.					
12	Sometimes video/technology may assist lectures, but it cannot replace a good teacher.					
13	Lectures create role models among teachers for students.					

For the final questionnaire a Google form (Google, California) was created, and the link was sent to students to their email IDs. All students eligible were included in this initial round of emails. A participant information sheet and consent form were embedded on the first page, while names and email IDs were not asked to maintain anonymity. Data were analyzed in SPSS (IBM SPSS Statistic ^®^ version 25) (RRID:SCR_016479). The analyses included descriptive statistics and chi-square tests. Statistical significance was set at p<0.05.

## Results

We coded options on a Likert scale from 5 to 1 as SA (strongly agree-5), A (Agree-4), N (Neutral-3), D (Disagree-2), and SD (strongly disagree-1). Further, we divided the semesters into years to ensure uniformity and homogeneity, as shown in Table 2. There were a total of 1,545 students across all three institutions who received the questionnaire from students’ section. The link was sent to all students’ email. From JMC we received 300 respondents out of which 93 were males and 207 were females. From KMC we received respondents 677 out of which 380 were males and 297 were females. And from SMIMS we received 568 respondents out of which 171 were males and 397 were females. There were a total of 877 responses including responses from all three institutions and descriptive data is shown in Table 3. There were more female respondents than male (474 and 403 respectively). Responses from each year were comparable, and there was no significant difference, as shown in Table 3. ^34^

**Table 2.** Shows the division of semesters into years to have uniformity and homogeneity in statistical analysis. In medical, there are 9 semesters over 4 and half years, therefore, we grouped 3,4, and 5 semesters as year 2 because the same subjects are learned in this period of one and half years.

Nursing and physiotherapy	Medical
semester 1 and 2 as year 1	semester 1 and 2 as year 1
semester 3 and 4 as year 2	semester 3,4,5 as year 2
semester 5 and 6 as year 3	sem 6,7 as year 3
semester 7 and 8 as year 4	semester 8,9 as year 4

**Table 3.** Shows the total responses of students from each center and other characteristics.

	Jorhat Medical College	Kasturba Medical College	Sikkim Manipal Institute of Medical Sciences	Total
**Total Responses** (n=877)	201 (22.9%)	304 (34.7%)	372 (42.4%)	877
**Male:Female**	86:115	147:157	171:201	877
**Year 1**	58	77	95	230
**Year 2**	57	75	92	224
**Year 3**	54	67	86	307
**Year 4**	32	85	99	216

Table 4 shows question wise responses of students on the Likert scale. Question 1 (Q1) was about missing lectures for which 58.7% of the students agreed (or strongly agreed) they had, which is very large compared to disagree(10.9%) and strongly disagree(6.6%). Around 60% of students agreed (or strongly agreed) to genuinely missing lectures, as a response to Q 2. However, 65.6% of the students agreed that they were happy that online teaching was available. Despite this, answers to Q4 77.9% reinforced that the online mode of the lecture could not replace usual classroom lectures. In Q5, 87.6% students agreed (or strongly agreed) and expressed that the quality of the teacher, makes a good classroom lecture. Around 48.9% of students disliked a lecture where the teacher was either reproducing the book or confused about their teaching methods; 55.2% of students believed that it is better to shift to technology-based lectures only when the teacher is not capable of delivering a good lecture. This means that students find online mode as an alternate when teaching quality in the classroom is not good. Also, 81.3% of students eagerly wait for some lectures because of the teacher’s ability, this may show students seeing a role model in teachers. Only 23.2% of students thought that the online mode was sufficient and could be a replacement for classroom teaching. Further, 59.2% of students felt that classroom teaching is the best method of teaching-learning and it gives great impetus to strengthening lectures. In Q11, 87.8% of students agreed that lectures are nothing but the teacher’s ability to deliver, and its success depends on the quality of the teacher. Next, 83% of students felt that technology such as video streaming is just an adjunct to make lectures better, but it cannot suffice and replace the role of the teacher in the lecture. Also, 71.4% of students looked for role models in their teachers during lectures, which is a big factor to be analyzed.

**Table 4.** Shows specific responses in percentage to each question ranging from SA (Strongly agree), A (Agree), N (Neutral), D (Diasagree) to SD (Strongly disagree).

	SD	D	N	A	SA	Total
	N (%)	N (%)	N (%)	N (%)	N (%)	n
Q1	58 (6.6)	96 (10.9)	208 (23.7)	343 (39.1)	172 (19.6)	877
Q2	84 (9.6)	83 (9.5)	183 (20.9)	367 (41.8)	160 (18.2)	877
Q3	23 (2.6)	72 (8.2)	207 (23.6)	419 (47.8)	156 (17.8)	877
Q4	31 ( 3.5)	44 ( 5.0)	119 (13.6)	307 (35.0)	376 (42.9)	877
Q5	7 (0.8)	8 (0.9)	94 (10.7)	420 ( 47.9)	348 (39.7)	877
Q6	36 (4.1)	171 (19.5)	241 (27.5)	280 (31.9)	149 (17.0)	877
Q7	51 (5.8)	96 (10.9)	246 (28.1)	329 ( 37.5)	155 (17.7)	877
Q8	12 (1.4)	29 (3.3)	123 (14.0)	400 (45.6)	313 (35.7)	877
Q9	210 (23.9)	287 (32.7)	177 ( 20.2)	148 (16.9)	55 (6.3)	877
Q10	47 (5.4)	127(14.5)	184 ( 21.0)	389 (44.4)	130(14.8)	877
Q11	7 (0.8)	11 (1.3)	89 (10.1)	462 (52.7)	308 (35.1)	877
Q12	7 (0.8)	26 (3.0)	99 (11.3)	404 ( 46.1)	341 (36.9)	877
Q13	8 (0.9)	18 (2.1)	225 (25.7)	419 (47.8)	207 (23.6)	877

Table 5 shows that KMC Mangalore students had a statistically significant better perception of onsite lectures compared to other institutions (p=0.007). There was no significant association between the gender of students and choices for the lecture; both genders felt that lectures were important (p=0.715). All the students had a good perception in favor of lectures, and they felt that a large group of teachers is indispensable as per the items of the questionnaire (p=0.445). Across disciplines, there was no significant difference (p=0.635), and all healthcare students in medical, nursing, and physiotherapy had a good perception of classroom lectures.

**Table 5.** Shows different variables/determinants and their correlation with the level of perception. The questionnaire was a Likert scale and for ease of study, scores were divided into two. Strongly disagree, disagree, and neutral was combined to be called ‘poor perception in favour of lectures’, and strongly agree and agree were combined to get one score and to be called ‘good perception favour of lectures’. This was done to get a clear distinction between the perception of students to have or not to have lectures.

Variables/Determinants		Poor/Average perception	Good perception		Chi square value and p value
Place of study					
	Jorhat	113	88	201	
		56.2%	43.8%	100.0%	
	KMC	131	173	304	
		43.1%	56.9%	100.0%	
	SMIMS	196	176	372	
		52.7%	47.3%	100.0%	
	Total	440	437	877	
		50.2%	49.8%	100.0%	χ ^2^=9.977; p=0.007
Gender					
	Male	200	204	404	
		49.5%	50.5%	100.0%	
	Female	240	233	473	
		50.7%	49.3%	100.0%	
	Total	440	437	877	
		50.2%	49.8%	100.0%	χ ^2^=0.133; p=0.715
Year of study	1	108	122	230	
		47.0%	53.0%	100.0%	
	2	117	107	224	
		52.2%	47.8%	100.0%	
	3	111	96	207	
		53.6%	46.4%	100.0%	
	4	104	112	216	
		48.1%	51.9%	100.0%	
	Total	440	437	877	
		50.2%	49.8%	100.0%	χ ^2^=2.672 p=0.445
Discipline					
	Medical	311	298	609	
		51.1%	48.9%	100.0%	
	Nursing	56	56	112	
		50.0%	50.0%	100.0%	
	Physiotherapy	73	83	156	
		46.8%	53.2%	100.0%	
	Total	440	437	877	
		50.2%	49.8%	100.0%	χ ^2^=0.908; p=0.635

## Discussion

Our study showed that students value lectures as a preferred teaching-learning method and do not see it as replaceable through online lectures or any other form of learning. There were 13 questions that tested students’ perceptions in different ways, such as asking whether it was just their fear or anxiety of having moved out of campus overnight and detached from the usual form of learning on campus; the majority students still rated lectures as indispensable (59.2%) of students responding to question 10.

A question we considered when completing this study was is it human nature to underestimate something that is easily available? Students by default attend lectures and the COVID-19 era was an unwarranted blow that shifted them from campus life to the home set up. In this dire state, we believe they could introspect the superior method and the importance of regular lectures better, especially when it was no longer feasible. Lectures are not mere delivery of content, but students can see a dynamic teacher in the classroom. It illuminates soft skills, aspects of delivery styles, and often looks for a role model in one of the teachers, ^2^ which is not possible when lectures are arranged online. The shift to online lecture was not a choice; rather a hard decision and better than nothing in the face of COVID-19 and social distancing protocols. However, the COVID-19 era could be seen as a blessing in disguise to have brought introspection about the method of face-to-face classroom lectures and its utility.

Our results suggest that self-directed, web-based, and video-based learning can be an adjunct to in person lectures but not a direct replacement; they must be engraved in the curriculum with caution, and teachership should not lose its essence. The teacher is the director of learning and changing roles in the new era and increasing workload or a teacher gaining multiple roles could be solved by bringing more teachers on board rather than replacing lectures. ^18^

A previous landmark study identified 12 roles of teachers derived from three models and six broad areas underneath. It is not a lecture *per se*, which is disliked but the poor quality of lecturing. ^2^^,^^10^ Our results suggest that, if the lecture is just a reproduction of text material, large coverage with no take-home message, amorphous talking, unorganized content, lack of audio-visual aids, poor voice quality, and inaudible, a large amount of students seem to avoid it. The few available literature note suggest that lectures are the best in presenting information, explaining, provoking new thoughts, adapting a problem-solving approach, improving inquisitiveness, critical and rational thinking, and deductive reasoning in limited time to a large group and all from the experiences of a good lecturer, which cannot be found in any other method. ^19^^–^^22^

Lectures can be improved by structuring and sustaining the interest of students with a clear learning objective during the session and a high engagement process. ^23^ The key messages can be summarized at the end, and students may take notes or handouts may be provided. There are various skills of lecturing, including planning, set induction, effective narration, questioning, and effective use of audio-visual aid making them interactive to improve learning. ^23^^,^^24^ This also takes away the notion that lecturing is an inborn quality; rather it can be learned. ^10^^,^^25^ An effective method is to obtain feedback from students and peers with a positive intent. Lectures need not be replaced; they should be improved by new digital innovations. ^2^^,^^11^ The purpose of two published studies ^26^^,^^27^ was to determine the lecturers’ or students’ perceptions and their achievement between two learning cultures, the traditional and the flipped classroom. Changing from traditional to flipped has reportedly had a positive impact on students’ perception and achievement. The cost to achieve this will include greater effort and time in the development of resources, planning, and implementation of in-class activities. The authors ^10^^,^^11^ stated that a new learning environment helped lecturers achieve their learning outcomes and made the teaching-learning process more engaging, active, and student-centered. These are innovative methods that can be used as adjuncts and not replace lectures, such as flipping.

Another study ^28^ examined student perceptions of lecture videos used as a means to increase the available time for in-class problem-solving in a teaching and learning context. A portion of the face-to-face lecture were replaced with pre-recorded lecture videos, which were assigned as homework. The freed lecture period was used for additional in-class problem-solving development without sacrificing the theory and fundamental background. To assess the effectiveness of the format change, student perceptions were assessed through an anonymous online survey. It was administered after completion of the course. Student perception of the lecture video was used to increase the time for an in-class course on ‘problem-solving applications. 70% of respondents in this study approved video lectures but they did not compare to the efficacy of classsroom lectures rather they informed that they can see video lectures at their ease, faster way of covering the syllabus and escaping from question-answer interaction of classrooms.

Another study ^29^ related to higher education, stressed students’ ratings to evaluate and improve the quality of courses and professors’ instructional skills. It is concerned with the psychometric properties of the instructional skills questionnaire (ISQ), a new theory-based student-rating-of-teaching questionnaire with specific questions concerning lecturing skills. The ISQ was developed by authors of this study and was administered after a single lecture in this study. It serves as a formative feedback instrument for university professors during courses to assist, improve, and re-evaluate their skills as deemed necessary. The ISQ contains seven dimensions of the professor’s instructional skills and three student-specific (self-perceived) learning outcomes. In this study, ^13^ Dutch students from an array of 75 courses rated three 90-minute lectures (T1, T2, and T3) and their respective professors using this ISQ. In aggregate, 14,298 ISQ forms were used to rate 225 lectures. The form is about a set of questions that were developed for the students’ post-lecture feedback, while the lecturers were instructed to conduct interactive classes. We reiterate that lecture classes were assumed to be taken as interactive and interesting to sustain students’ focus on improving learning.

Another study ^30^ used web-based learning support, augmented by multimedia theory, comprising interactive quizzes, glossaries with audio, short narrative PowerPoint presentations (Microsoft, US), animations, and digitized video clips. Here, 81 % of the students valued interactive multimedia learning only as an adjunct but were not yet ready to abandon the traditional face-to-face mode of lectures. This finding contributes to an understanding of how web-based resources can be supplementary, but not a substitute for face-to-face lectures. Similar findings were shown in our study, where students felt web-based learning only as a support medium for lectures.

Online learning is not a new concept. However, until a decade ago, these were mainly print-based and postal department-dependent. In India and the so-called third world nations on the threshold of emerging as developed ones, the technical aspect and resource limitations side-tracked us from web-based learning, in preference to traditional lectures. ^31^ Despite its immense potential, online teaching was not intended to be used exclusively, this was changed due to the spread of the pandemic, which opened the flood gate.

The authors reinforce that to ensure quality to be retained during online learning, the principles that had been identified during traditional classroom teaching should be incorporated into the online milieu. When we accessed old literature/journals, they mentioned that technology-based learning was not a replacement for the lecture because the very principles of web-based learning were configured in traditional classrooms. ^32^^,^^33^

Lectures are at least as effective as other teaching methods for imparting information and explanations. Intention, transmission, and output are the basis for model lecturing. The key skills of preparing lectures, explaining, and varying student activities may be derived from the models proposed in the published literature. ^2^

## Conclusions

Classroom teaching has evolved through experiments of the past. There is still scope for improvement in different aspects, but it should not be discarded in favor of online teaching. This small but sincere study again strongly reasserts its position and acceptance among contemporary students.

This study has limitations, and its generalizability will depend on context. This study has been done when students were suddenly shifted from On-campus education to online learning amidst COVID pandemic. Students might be having many questions about reopening of the colleges, impact of pandemic etc. which might influence their responses.

This study has a unique strength that it tried to find importance of classroom lectures and large group teaching amidst the crisis whereas most of the studies published in this era focused on Online learning.

## Data availability

### Underlying data

OSF: Perception of Health care students towards lectures as a Teaching-learning method in COVID era - A multicentric study from India. DOI: https://doi.org/10.17605/OSF.IO/FUEW4 ^34^

This project contains the following underlying data:

- All in One.xlsx (raw Likert scale data and key)

### Extended data

OSF: Perception of Health care students towards lectures as a Teaching-learning method in COVID era - A multicentric study from India. DOI: https://doi.org/10.17605/OSF.IO/FUEW4 ^34^

This project contains the following extended data:

- Data analysis final.doc (Data used in analysis).
- INITIAL QUESTIONNAIRE.docx (A word document containing the full set of questions used in the final questionnaire)

### Reporting guidelines

OSF: Perception of Health care students towards lectures as a Teaching-learning method in COVID era- A multicentric study from India. DOI: https://doi.org/10.17605/OSF.IO/FUEW4 ^34^

This project contains the following reporting guidelines:

- STROBE for lecture stody.docx
