# Supplementary material for: Perception of health care students towards lectures as a teaching and learning method in the COVID era - A multicentric cross-sectional study from India
Source: F1000Res. 2022 Oct 10;11:665. Originally published 2022 Jun 16. [Version 2] doi: 10.12688/f1000research.110100.2 (PMC9623191; doi:10.12688/f1000research.110100.2)
Supplement: Peer review history

# Peer review: Reviewer response for version 2

**DOI:** 10.5256/f1000research.139590.r152962

**Published:** 2022-10-31

**Authors:** Jyotsna Rimal

**Affiliations:** 1 Department of Oral Medicine and Radiology, BP Koirala Institute of Health Sciences, Dharan, Nepal

All the comments have been addressed. The manuscript can be accepted for indexing.

Is the work clearly and accurately presented and does it cite the current literature?

Yes

If applicable, is the statistical analysis and its interpretation appropriate?

Yes

Are all the source data underlying the results available to ensure full reproducibility?

Yes

Is the study design appropriate and is the work technically sound?

Yes

Are the conclusions drawn adequately supported by the results?

Yes

Are sufficient details of methods and analysis provided to allow replication by others?

Yes

Reviewer Expertise:

NA

I confirm that I have read this submission and believe that I have an appropriate level of expertise to confirm that it is of an acceptable scientific standard.

# Peer review: Reviewer response for version 1

**DOI:** 10.5256/f1000research.121675.r151525

**Published:** 2022-09-29

**Authors:** Tapasya Karemore

**Affiliations:** 1 Department of Oral Medicine and Radiology, V.S.P.M Dental College, Nagpur, India

It is a very novel study. COVID brought many questions to the thought of health educationists. During COVID platform of education got shifted to Online. But what about Lectures? Whether students are really satisfied with online. Pertaining to this issue many educationists published papers which were more of narrative and hardly inquired about the perception of students.

 Biggest positive point of this study is taking three different health profession students, medical, nursing and physiotherapy across three geography, three reputed institutions in India, and explored students' perception through a survey using a questionnaire. The questionnaire was validated well, as per standard guidelines. Data was collected with confidentiality and then most appropriate statistical tools were used. Data is well presented. Sample size is very good. Discussion is well written. Authors are reputed and related to education. Conclusion is well drawn. This study is well at time and requires early publication before it gets its impact among readers and policy makers. Lectures and Large group teaching can not be replaced by anything, it can be made better using innovative ways to sustain students attention. This point is well written by authors.

Is the work clearly and accurately presented and does it cite the current literature?

Yes

If applicable, is the statistical analysis and its interpretation appropriate?

Yes

Are all the source data underlying the results available to ensure full reproducibility?

Yes

Is the study design appropriate and is the work technically sound?

Yes

Are the conclusions drawn adequately supported by the results?

Yes

Are sufficient details of methods and analysis provided to allow replication by others?

Yes

Reviewer Expertise:

Education, Medical education, Oral Medicine and Radiology, Dental Education Technology

I confirm that I have read this submission and believe that I have an appropriate level of expertise to confirm that it is of an acceptable scientific standard.

# Peer review: Reviewer response for version 1

**DOI:** 10.5256/f1000research.121675.r142261

**Published:** 2022-08-01

**Authors:** Jyotsna Rimal

**Affiliations:** 1 Department of Oral Medicine and Radiology, BP Koirala Institute of Health Sciences, Dharan, Nepal

The manuscript brings one of the most debated topic of large group physical lecturing Vs online teaching to its highlight. This work is important and brings richness of data through its multicentric design. However there are few minor corrections needed to bring it to indexed form. Please see the attached pdf for further comments.

Is the work clearly and accurately presented and does it cite the current literature?

Yes

If applicable, is the statistical analysis and its interpretation appropriate?

Yes

Are all the source data underlying the results available to ensure full reproducibility?

Yes

Is the study design appropriate and is the work technically sound?

Yes

Are the conclusions drawn adequately supported by the results?

Yes

Are sufficient details of methods and analysis provided to allow replication by others?

Yes

Reviewer Expertise:

Oral health, assessment, teaching learning, curriculum, quality assurance, problem based learning, faculty development programs

I confirm that I have read this submission and believe that I have an appropriate level of expertise to confirm that it is of an acceptable scientific standard, however I have significant reservations, as outlined above.

# Response

**Published:** 2022-10-07

**Authors:** VIJAY PRATAP SINGH

**Affiliations:** MANIPAL ACADEMY OF HIGHER EDUCATION, India

Reviewer’s comments

**Changes made**

 Inference drawn in the introduction itself. The facts followed by evidence based inference would be better

**Response:** removed and reframed

 No method is good or bad however.....

**Response: **No method is good or bad however any method should be evaluated from the perspective of the situational fulcrum.

 took this opportunity

**Response: **We took this opportunity to find out students’ perception toward lectures…..

 In order to avoid bias, the research design was aimed at complete enumeration, blinding and voluntary nature.

**Response: **done

 This and the subsequent sentence not needed.

**Response: **done

 should be strongly disagree not otherwise

**Response: **done

 typographic error

**Response: **done

 shows not needed

**Response: **done

 This is an important table. This table's result should be inferred and discussed in the discussion section. For eg: what could be the inference from the first question?

**Response: **done

 shows not needed

**Response: **done

 The column heading is missing

**Response: **done

 just the way question number 10 is addressed...other questions need to be discussed and inferred

**Response: **done

 how did the study show 'self directed learning could be an adjunct?

**Response: **This is an extrapolation based on the fact that how some Institutions want to replace Lectures by these methods. Kindly accept

 Since, the study findings support lectures, 'at least' seem inappropriate here

**Response: **done

 should have been ' which might have influenced.....'

**Response: **done

 The conclusion is to recommend lectures so the last part of the sentence seem irrelevant (Whereas most of the studies......on online learning)

**Response: **done
